# Divergence in Defence against Herbivores between Males and Females of Dioecious Plant Species

**DOI:** 10.1155/2012/897157

**Published:** 2012-12-23

**Authors:** Germán Avila-Sakar, Cora Anne Romanow

**Affiliations:** Department of Biology, The University of Winnipeg, Winnipeg, MB, Canada R3B 2G3

## Abstract

Defensive traits may evolve differently between sexes in dioecious plant species. Our current understanding of this process hinges on a partial view of the evolution of resistance traits that may result in male-biased herbivory in dioecious populations. Here, we present a critical summary of the current state of the knowledge of herbivory in dioecious species and propose alternative evolutionary scenarios that have been neglected. These scenarios consider the potential evolutionary and functional determinants of sexual dimorphism in patterns of resource allocation to reproduction, growth, and defence. We review the evidence upon which two previous reviews of sex-biased herbivory have concluded that male-biased herbivory is a rule for dioecious species, and we caution readers about a series of shortcomings of many of these studies. Lastly, we propose a minimal standard protocol that should be followed in any studies that intend to elucidate the (co)evolution of interactions between dioecious plants and their herbivores.

## 1. Introduction

Sexual systems in angiosperms range from hermaphroditism (monomorphic populations of plants with bisexual flowers) to dioecy (dimorphic populations of male and female individuals) and include almost all imaginable combinations and gradations (Table 1; [1, 2] and references therein). Such remarkable diversity of sexual systems has perplexed naturalists and evolutionary biologists for a long time [3–6]. The evolution of dioecy from a hermaphroditic ancestor has been particularly difficult to understand because the invasion and maintenance of unisexual mutants in a population of hermaphrodites require that the loss of fitness resulting from the loss of one sexual function be compensated by increased fitness gains through the remaining sexual function of the unisexual mutant [7, 8]. This requirement seems very restrictive, and therefore considerable effort has been devoted towards understanding the conditions under which dioecy can evolve [5, 8–18]. In contrast, the evolution of sexually dimorphic traits following the evolution of dioecy (successful establishment of only two reciprocal unisexual morphs in a population) has received less attention. Consequently, our current understanding of the evolution of sex-related traits ultimately leading to morphological or physiological differences between unisexual morphs (i.e., sexual dimorphism or secondary sexual traits) is still limited, despite recent advances and excellent syntheses on the topic [5, 6, 19–21]. This paper focuses on one set of traits subject to becoming sexually dimorphic upon the evolution of dioecy: those traits that provide plants with defence against herbivores. 

### 1.1. Herbivory and the Evolution of Dioecy

**Figure 1:**
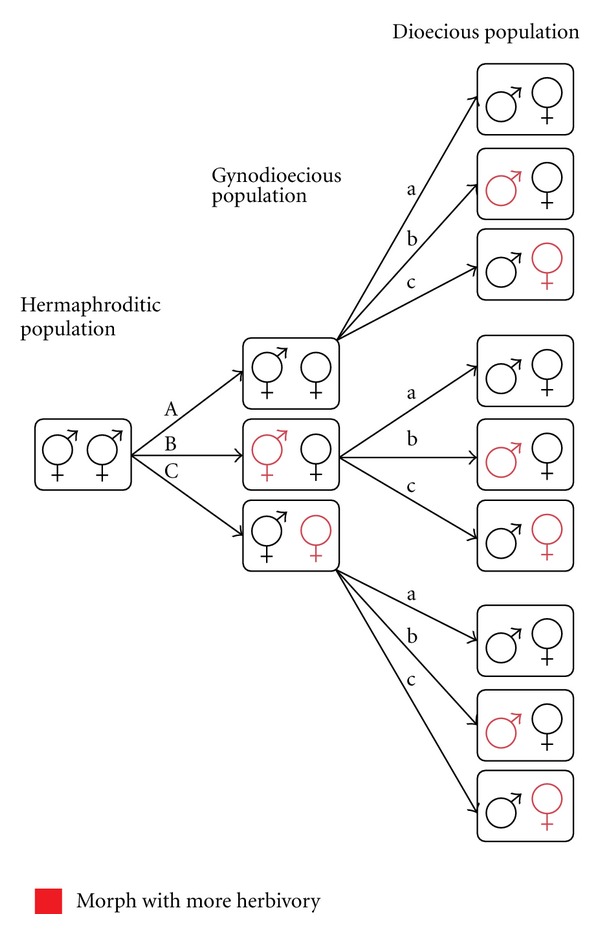
Possible scenarios of the inception of morph- or sex-biased herbivory in the evolution of dioecy via the gynodioecy pathway. Symbols represent hermaphrodite, male, or female morphs in a population (rectangle). Arrows represent evolutionary pathways between populations with different sexual systems. The first step in the pathway is the transition from a hermaphroditic to a gynodioecious population by the successful invasion of females (left-most set of arrows). The second step in the pathway is the transition from a gynodioecious to a dioecious population following the successful invasion of males and the disappearance of hermaphrodites (right-most arrows). Letters indicate different evolutionary paths.

Sex-biased herbivory may be one of the selective pressures conducive to the evolution of dioecy, and it can also be a consequence of sex-specific selection on patterns of resource allocation in dioecious species. Considering only the gynodioecy pathway of the evolution of dioecy, we can think of three possible scenarios regarding the role of herbivory in each of the two steps involved in this pathway (Figure 1). The first step in the gynodioecy pathway to dioecy is the successful establishment of females (male-sterile mutants) in a population of hermaphrodites, thus resulting in a gynodioecious population. As mentioned above, this step requires that females compensate for the fitness loss incurred with the loss of the male function. The reallocation of resources freed from the male function towards defence may contribute towards fitness compensation if increased defence results in greater fitness for the females [9]. Increased defence may result in lower herbivore damage on females than on hermaphrodites (Figure 1, path B). However, this is not the only possibility. Defence may be achieved through resistance: traits that reduce the rate of herbivore attack such as low nutritional content of tissues (particularly, N content), secondary metabolites, trichomes, cutin, waxy cuticles, lignin, and volatiles that attract natural enemies of herbivores [22]; and also through tolerance: traits that mitigate the negative effects of damage on fitness, including higher or lower growth rates, mobilization of stored resources, and activation of apical meristems [23]. If females reallocate resources to tolerance traits, they could be the morph with greater herbivore damage (Figure 1, path C).

The second step in the gynodioecy pathway to dioecy is the successful establishment of male individuals (female-sterile mutants) in a gynodioecious population followed by the loss of the hermaphroditic morph, thus resulting in a dioecious population. Upon the evolution of two unisexual morphs, defensive traits may evolve differently in each sex and eventually become sex linked [84–86]. The particular way in which defensive traits diverge between sexes will depend on the costs and benefits derived from the specific pattern of resource allocation to growth, reproduction, and defence in each sex. Currently, it is thought that females generally evolve greater resistance than males (see the following; Figure 1, path b).

 This paper focuses on the origin of sex-biased herbivory in dioecious species. Therefore, we will not delve into morph-biased herbivory in gynodioecious species, which would be the topic of a different essay. However, we do recognize that sex-biased herbivory—indicative of sexual dimorphism in resistance against herbivores—is likely related to morph-biased herbivory in the ancestral gynodioecious population from which it evolved (Figure 1, path B-b). Thus, male-biased herbivory may have its origin in a gynodioecious population where hermaphrodites (functionally, the male morph) bear greater levels of herbivory than females. 

### 1.2. Male-Biased Herbivory

The above view for the origin of greater resistance against herbivores in females is based directly on the principle of allocation: resources freed from the male function are used for the female function, growth, and defence. In contrast to this view, the finding of male-biased herbivory in dioecious populations has been explained on the basis of sex-specific selection of resistance traits, where the main difference between sexes that drives the sex-specific selection is the cost of reproduction. In this alternative view, female individuals of dioecious species are expected to have lower herbivory levels than males because the higher cost of reproduction of females confers a selective advantage to females with traits that reduce herbivore attack [66]. The logic of this argument is as follows: since females invest more in reproduction than males, they are left with a smaller pool of resources for growth and therefore must grow more slowly than males [20, 87]. According to the resource availability hypothesis, the fitness cost of losing tissue to herbivores is greater for plants that grow more slowly, thus favouring the evolution of increased defence against herbivores in slow-growing plants [88]. Since females tend to grow more slowly, they should be better defended against herbivores than males [88]. Consequently, dioecious species should experience male-biased herbivory. We must note that the argument for greater defence levels in females is usually understood in terms of resistance, but it could also be interpreted in terms of tolerance, in which case the predictions of sex-biased herbivory would be the opposite (Figure 1, path c), as developed below.

The first review of the empirical studies on the topic of sex-biased herbivory concluded that “males are more likely than females to be preferentially used by herbivores” and suggested that male-biased herbivory was widespread among dioecious species [72]. The authors, however, recognized that sex-biased herbivory was by no means a unanimous finding across all the dioecious species examined to that date, and that the relative susceptibility of each sex to herbivory could be influenced, among other factors, by fluctuations in ecological tradeoffs between functions (rather than evolutionary changes in patterns of allocation), such as phenological changes in resource allocation to reproduction and growth [75, 89]. Therefore, the life stage at which damage measurements are taken can determine whether a study concludes that herbivory is sex biased or not. 

In addition, Ågren et al. cautioned against publication bias, whereby studies that found differences between genders could be more likely to be published than those that did not; and taxonomic bias, an overabundance of studies from certain genera or families. In this instance, the taxonomic bias is correlated with an ecological bias for studies of temperate species, despite dioecy being more prevalent in tropical ecosystems [90]. Ågren et al. called for future studies that (1) examine the causes of differential palatability between males and females, (2) measure the fitness consequences of natural levels of herbivory in both sexes, and (3) determine whether herbivore pressure can actually cause adaptive changes in tissue palatability. In addition, they urged for broadening the taxonomical scope of the studies. In spite of such encouragement, there still is a paucity of studies that address these issues.

More recently, sex-biased herbivory in dioecious species was tested by means of a meta-analysis of 33 studies encompassing 30 species, 19 of which were previously included in Ågren et al.'s 1999 review [73]. The authors tested for publication bias and found it to be minimal. However, they did not emphasize other shortcomings of the dataset and concluded that male-biased herbivory in dioecious species is a rule.

Here we propose alternative evolutionary scenarios that could result in female-biased herbivory or lack of intersexual differences in herbivory levels. We invite the reader to reconsider the evidence for male-biased herbivory in dioecious plants and recommend a standard protocol for evolutionary-ecological studies of sex-biased herbivory in dioecious species that addresses the shortcomings listed below. We contend that taking for granted the generality of male-biased herbivory in dioecious species is hampering our progress in this field. 

## 2. Critique of Theory: Evolutionary Scenarios

 While male-biased herbivory has been explained as a consequence of sex-specific selection of resistance determined by the cost of reproduction of each sex, few of the reviewed studies (Table 2) are actually placed within such evolutionary context. J. Lovett-Doust and L. Lovett-Doust [44] were the first to argue, citing Charnov [91], that an evolutionary divergence between sexes in resource allocation patterns could result in sex-biased resistance against herbivory. Danell et al. [57] based their expectation of male-biased herbivory on sexual selection: it may be more advantageous for males to invest less in resistance and more in reproduction when exposed to a cyclic herbivore compared to a noncyclic herbivore because males will lose fitness only once every four years, and their reproductive output during years of little or no herbivory will more than compensate for the fitness lost to herbivores on the heavy herbivory years. The above explanation would hold only to the extent that there are no carry-over effects from one year to another. 

More recently, McCall [83] cited Bierzychudek and Eckhart [92] and Delph [87] in support of the claim that the reproductive output of females is more limited by resources than that of males. However, while there may be ample evidence that reproductive allocation is generally greater for the female function, it is possible that, upon the separation of sexes, physiological mechanisms involved in resource acquisition and allocation evolve in such way as to minimize the differences in reproductive effort between sexes. For example, in *Ilex glabra*, sexes do not differ in total reproductive biomass produced in a growing season because the greater unitary investment in pistillate flowers and fruit development is negated by the sevenfold greater flower production in males [75]. Given the importance of the tenet of greater female reproductive allocation for the expectation of male-biased herbivory, all studies of sex-biased herbivory should test for intersexual differences in reproductive allocation or provide a reference to an empirical study that demonstrates such differences for the species in question. In the measurement of reproductive allocation, particular attention must be paid to obtaining reliable estimates of male reproductive output (pollen production), which presents its own logistical difficulties. In addition, resource expenditure in pollinator attraction needs to be considered, as this is another expenditure related to reproduction that may differ between sexes.

 In essence, the presumed chain of evolutionary events that lead to male-biased herbivory in dioecious plants stems from the reallocation of those resources freed upon the loss of a sexual function in unisexual mutants towards defence. In most studies, defence has been equated to resistance. However, defence may also occur through tolerance [22, 93]. In comparison to resistance traits, tolerance traits have been more elusive. The capacity to store and mobilize carbohydrates, the presence of meristems and the capacity to activate them in response to damage have been proposed as tolerance traits [23]. Growth rate has also been proposed to influence tolerance, although it is controversial whether high or low growth rates favour tolerance [94–98]. A recent model shows that plants with low growth rates are more tolerant to herbivore damage [99]. This model also shows that plants that can change their growth rate positively in response to damage will tolerate damage better than those with a different response in growth rate. The activation of meristems and mobilization of resources in response to the loss of tissue are two well-documented responses [100, 101] that could contribute to increased plant growth rates in response to damage. Thus, according to this model, if females grow more slowly than males because of their greater allocation to reproduction, then females should be more tolerant to herbivory than males. This response was observed in *Urtica dioica *subjected to clipping of the stem apex [102]. Whether this prediction necessarily implies that females should be less resistant than males is not clear at this point since there is increasing evidence that these two modes of defence are not necessarily mutually exclusive [99, 103, 104].

 There is one other possibility that has not been emphasized enough in the proposed models of the evolution of defence in dioecious species: while one possible consequence of a greater allocation of resources to reproduction in females is reduced allocation to growth, it is also possible that the main reduction in allocation is to defence. In this case, there would be no detectable detriment to growth. Consequently, female plants would suffer more damage (if they are less resistant; Figure 1, path c) or greater fitness losses (if they are less tolerant) compared to males. Greater damage in females could lead to fewer, more spaced reproductive events or greater interannual variability in reproductive output either directly through a decrease in the availability of resources for reproduction or indirectly through a decrease in pollinator visitation rates due to a lack of resources needed for floral display or nectar production [75, 105–108]. Fewer resources available for reproduction could also pose a selective pressure to become choosier about their mates, which may lead to increased fruit or seed abortion [75, 109, 110]. 

 Evolutionary changes in the rate of resource acquisition in female individuals may occur through increased photosynthetic rates, canopy area, rates of mineral nutrient uptake, as well as greater branching of roots, and enhancement of mycorrhizal associations [19]. A greater rate of resource acquisition in females would decrease the relative differences in costs of reproduction between sexes: the sex with the greater resource demand for reproduction would have an increased capacity to garner resources. Under such scenario, the life-time cost of reproduction at the individual level would be equal between sexes, thus eliminating the source of inequalities in the patterns of allocation between males and females. In summary, nothing dictates that there is only one evolutionary pathway regarding changes in the patterns of resource allocation among reproduction, growth, and defence following the evolution of unisexuality (Figure 1).

 Alternatively, a stage in which female individuals have heavier damage levels because of resource limitation for resistance may be a transient evolutionary stage prior to the invasion of mutants whose greater defence levels are attained at the expense of growth. In this case, we should observe female-biased herbivory in younger dioecious lineages and male-biased herbivory in those lineages in which there has been enough time for selection to reshape the patterns of resource allocation to reproduction, defence, and growth. We should be able to test this by means of relative dating of lineages with male- or female-biased herbivory.

Similarly, as long as there has not been selection on prereproductive growth rates following the evolution of unisexuality, we should not see differences in growth rates or other physiological vegetative traits between males and females before their first reproductive event. It is difficult to test this prediction without reliable morphological or genetic markers that allow juveniles to be sexed so that their performance can be compared on the basis of sex. Some sex-linked markers may be, effectively, sex-related traits expressed before the onset of reproduction. Whether the presence of these markers implies the existence of sex chromosomes is still an area in need of further investigation [84, 85, 111–113]. 

 In short, without fitness gain curves for each sex, it is difficult to predict accurately which sex should evolve greater resistance against herbivores and whether we should expect or not male-biased herbivory in dioecious species [91]. In fact, we need fitness surfaces in order to include the effects of reductions in leaf area caused by herbivory. Moreover, the fitness surfaces should account for the short- and long-term responses of plants in terms of changes in photosynthetic rates, reallocation of resources to shoot or root, activation of meristems, and delays in phenology or shortening of life span brought about by herbivore damage [95, 99]. 

 It has not escaped our attention that the evolution of defence in gynodioecious species can be approached from a similar perspective to the one presented above for dioecious species [12]. It is important to consider that some of gynodioecious species may be in evolutionary transition to dioecy while others are not [112, 114]. Another important difference with dioecious species is that, in gynodioecious plants, the morph that performs the male function—the hermaphrodites—may have a greater cost of reproduction because of the expenditure of resources on two sexual functions. Does this mean that hermaphrodites would be less resistant (Figure 1, path C) or grow more slowly and evolve greater resistance (Figure 1, path B)? Clearly, making predictions with respect to gender dimorphism in defensive traits for bisexual conditions along the gradation from monoecy to dioecy is not straight forward.

## 3. Critique of Datasets Used to Conclude Male-Biased Herbivory

The collection of studies cited in the reviews of herbivory in dioecious species [72, 73] has, as a group, important shortcomings that weaken the conclusion of male-biased herbivory as a generality in dioecious species. The main shortcomings are (1) the taxonomic bias of the sample of species studied; and (2) failure to test for or provide references of empirical studies of intersexual differences in (a) resistance traits—the purported cause of the intersexual differences in herbivory levels; (b) growth rates—the purported cause of the intersexual differences in resistance to herbivore attack; and (c) reproductive effort—the purported cause of the aforementioned intersexual differences in growth rates. These deficiencies had been pointed out earlier [72, 89], but judging by statements included in the introduction or discussion of many of the papers published after the 1999 review, such caveats have not been considered to their full extent, and many authors take for granted either the generality of male-biased herbivory in dioecious species or its expectation without reference to any theoretical context. 

### 3.1. Taxonomic Bias

Cornelissen and Stiling's meta-analysis of sex-biased herbivory includes 30 species, 28 of which are angiosperms. Focusing only on angiosperms, 13 of the 28 species were not considered previously in Ågren et al.'s review (Table 2). These 30 species represent a total of 20 genera, 18 families, and 10 orders. Nine of those species belong to the same genus: *Salix*. Adding to those *Populus tremula*, the species in the Salicaceae represent one-third of all species considered for the meta-analysis. Such distribution contrasts greatly with the taxonomic distribution of dioecy in 14,620 species, 959 genera, 157 families, and 36 orders [90]. Of the four dioecious genera (consisting of solely dioecious species) with most species (400), only *Salix* has been studied. *Pandanus*, *Diospyros*, and *Litsea*, with 700, 500+, and 400 species, respectively ([115]; S. Renner, University of Munich, unpublished data) have not been studied for sex-biased herbivory yet. Clearly, we need to direct our research efforts to the most understudied orders and families if we want to arrive at generalizations regarding the biology of dioecious species, and particularly the influence of herbivores in their ecology and evolution.

In addition to this taxonomic bias, a critical reexamination of that list of species casts serious doubt on the conclusion that male-biased herbivory is a rule in dioecious species: only 13 of those species were reported invariably to have male-biased herbivory. This list includes three *Salix*, two *Freycinetia*, and two species for which evidence of male-biased herbivory has not been published: *Hippophae rhamnoides *and *Rumex acetosa*. (In fact, male-biased herbivory in *Myrica gale*—not included in these 13 species—is also anecdotal.) Greater herbivore damage on females is reported for four species, while the rest show either no intersexual differences (16), or variation in the result, depending on different factors (species of herbivore, kind of herbivore, tissue damaged, time of year, phenological or ontogenetic stage, etc.). Moreover, it is possible that the results for those 13 species would show variation with either population or site, had these factors been studied.

### 3.2. Differential Growth between Sexes

Perhaps the most serious problem with several studies of herbivory and dioecy has been the failure to make the connection between sex-biased herbivore damage and intersexual differences in growth rate, precisely because the latter is the purported cause of the former. Of the 30 species of angiosperms in Table 2, either growth rate or a surrogate variable for growth (e.g., shoot length) was measured only in 21 species. Males grew faster in six species, females in two, no difference between sexes was detected in six species, and three species showed variable results. It must be noted that the same number of species shows no difference between genders as those that show greater growth rate in males. Considering solely the 13 species that invariably showed male-biased herbivory, only two show a greater growth rate in males: *Acer negundo* and *Hippophae rhamnoides*. However, as the evidence for male-biased herbivory in *H. rhamnoides* is anecdotal, we are left with only one species for which growth rate was measured in the same study as herbivore damage: *Acer negundo*. 

### 3.3. Differential Reproductive Effort between Sexes

Only 12 of the 30 species listed were assessed for intersexual differences in reproductive allocation in terms of reproductive effort (the proportion of biomass or other currency devoted to reproductive structures relative to the total biomass or expenditure in the selected currency of an individual). Reproductive effort was greater in females of 10 species and in males for the other two species. In some species, reproductive effort was measured during flowering, but allocation to fruit production was not considered (e.g., *Silene dioica*). In those cases, we are left with an incomplete picture of reproductive allocation, and we can only join the authors in speculating whether species of the same genus have similar patterns of reproductive allocation.

The only species that have been assessed for foliar damage, growth rate, and reproductive allocation in the same study are *C. tepejilote*,* B. halimifolia*,* I. glabra, N. psychotrioides*, and *R. alpinus* (Table 2). These studies clearly made the chain of causal connections from sex bias in reproductive allocation all the way to sex bias in some resistance traits (except for *C. tepejilote *and* I. glabra*), and, as a consequence of the latter, sex bias in levels of damage. The study on *R. acetosella* at least established the connection between damage and growth [44]. The study of *R. alpinus *went even further, comparing these attributes between pre- and postreproductive plants, and thus emphasizing that the root of the differences in growth rates, resistance traits, and leaf damage is in the patterns of reproductive resource expenditures [63, 116].

In some species, reproductive allocation, growth rate, and/or resistance were reported after the initial publication of sex-biased herbivory. However, even with these studies, the number of species for which we have a more complete picture of the causal links amongst these attributes remains low: nine more species (*C. alternans, B. dracunculifolia. A. canescens, R. acetosella, S. caprea, S. cinerea, S. lasiolepis, S. sericeae,* and *H. rhamnoides *[?]; Table 2) now have published data for damage and growth rate, bringing the number of species in this situation to 15. Two more species, for a total of three, now have data on damage, growth rate, and reproductive allocation (*C. alternans, S. dioica, *and* N. psychotrioides*). Two more species now have data on reproductive allocation, growth rate, and resistance apart from herbivore damage, for a total of four species with all four variables measured (*A. triphyllum, B. halimifolia, L. benzoin*, and *R. alpinus*).

In summary, the majority of studies on the topic of sex-biased herbivory have neglected the purported causal connections between bias in reproductive allocation, differential growth rate, resistance, and herbivore damage. Also, some authors seemed to confuse theoretical expectations with empirical evidence of greater female reproductive allocation: while Lloyd and Webb [20] argue convincingly for the expectation of greater reproductive effort in females, they provided empirical evidence only for *Rumex acetosella*, citing Putwain and Harper 1972 [117]. Therefore, Lloyd and Webb's excellent paper cannot be cited as solid empirical evidence of greater reproductive effort in females. Lastly, anecdotal evidence should be taken with great caution and always flagged as such until data are published (see entries marked “?” in Table 2). 

Using the search terms herbiv* and dioec* for entries between January 1998 and May 2012 on the Web of Science, we found nine studies encompassing 14 species that were not included in either of the previous reviews of the topic. Of these, only the study on the three species of *Chamaedorea* palms measured reproductive allocation, growth rate, resistance, and herbivore damage (Table 3; N.B.: one of these species had been studied before: *C. alternans* = *C. tepejilote*). Only one other study measured damage and reproductive allocation (*Sclerocarya birrea*, Table 3). Similarly, growth rate was assessed in only one other species (*Salix arctica*). The taxonomic breadth of the studies of herbivory in dioecious species increased only by one family (in an unplaced order of the Euasterids I). The general lack of consistency in the level of detail and the variables that have been measured in all these studies could be addressed if researchers interested in this topic followed a minimally standardized protocol. 

## 4. Future Directions: Standardized Protocol and Broadening of Species Studied

 New studies must clearly allude to the theoretical framework from which the prediction of sex-biased herbivory levels (resistance) stems—resource allocation theory, in particular, sex allocation. The claim that male-biased herbivory is expected because it has been reported as a pattern, whether implicit or explicit, lacks heuristic value because it does not address the causes of such pattern. Moreover, a plethora of factors may modify the expected pattern, as shown above.

 Clearly, we need to increase the taxonomic breadth of the studies of herbivory in dioecious species. There are several ways to achieve greater taxonomic representation. We could direct our attention to those families with the greatest number of dioecious species or those with the greatest proportion of dioecious species. The first alternative will miss families with low species richness that may have a high proportion of dioecious species. The second method will miss families with high species richness but low proportion of dioecious species. One possible compromise is to focus our studies on the families with the greatest number of dioecious species among those with a large proportion of dioecious species, for instance, 50% or more (Table 4). So far, we have studied only 2% of the dioecious species in the most studied family (Salicaceae). If we took that as a target, we would have to study about 155 species for the 30 most dioecious species-rich families of angiosperms. However, by this method we would include only one species per family for many families, thus failing to achieve adequate representation of those families. In addition, we must consider that the conditions that determine sex-biased herbivory can change with habitat, and therefore some species may need to be studied in several habitats. 

 In addition to the taxonomic bias, there is a preponderance of studies of woody plants. While this is understandable because most dioecious species are woody, we should strive for representation of the herbaceous component. With increased research on herbaceous dioecious species, we can address the influence of life history traits on the evolution of dioecy and defence.

 Lastly, we propose that all studies aimed at assessing whether herbivory levels differ between sexes and whether these differences are a consequence of differential growth rates (in turn resulting from differential allocation to reproduction) should conduct, at least, the following measurements and observations: (1) levels of herbivory, measured as precisely as possible (preferably for more than one growing season in perennials); (2) species of herbivores responsible for most of the damage; (3) growth rates, measured either as RGR for whole individuals or from increments in branch length or leaf production; (4) reproductive allocation, measured both as the number of reproductive structures (flowers and pollen production for males, flowers, and fruits for females), and also as reproductive effort (the proportion of individual or shoot biomass allocated to reproduction, and when possible N and P allocation to reproductive structures); and finally (5) the most important resistance characters that could be influencing the levels of herbivory and measure them quantitatively. In addition, these studies could add an experimental component in which plants are damaged at least at the highest rate seen in the surveys of natural damage, so as to measure tolerance to herbivory as well as resistance [75, 102]. Ideally, damage should be performed by placing natural herbivores on the plants because mechanical damage does not necessarily elicit the same physiological responses as herbivore damage [118]. Also, these studies should consider that resistance and tolerance may vary both with ontogeny and with respect to other reproductive phenology because the acquisition and expenditure of resources vary at different stages of development and life history [119–121]. We must reiterate that other authors have emphasized the need to address several of the points outlined above. It is our hope that future studies take these recommendations seriously so that we have to assume and speculate less, and we have empirical data to further our understanding of the evolution of defence in dioecious species.

## 5. Conclusions

 The study of the evolution of sex-biased herbivory is hampered by the notion that male-biased herbivory in dioecious species is a rule. We have shown that the evidence used to support this conclusion has important shortcomings. We have presented other possible evolutionary outcomes with regards to sex-biased herbivory in the transition from hermaphroditic populations to dioecious ones. We have also discussed how these different outcomes can be predicted under different theoretical assumptions. Therefore, future studies of herbivory in dioecious species should be based on a clear theoretical framework. In particular, we urge that all new studies of herbivory in dioecious species include assessments of reproductive allocation, growth rates, and resistance traits deemed to differ between sexes and, therefore, determine sex-biased herbivory. In addition, tolerance should also be considered as a potentially important defence mode that can vary between sexes. In this manner, we should be able to explain better the results of any given study. The advancement of our knowledge about sex-related defence in plants should help us gain a better understanding of the evolution of sex-related traits in general.

## Figures and Tables

**Table 1:** Terminology for flowers and sexual systems.

Term	Description
Flowers	
Pistillate	Unisexual flower with functional pistils only (female flower; may have vestigial, sterile stamens (staminodia))
Staminate	Unisexual flower with functional stamens only (male flower; may have vestigial, sterile pistils (pistilodia))
Bisexual, perfect	Bisexual flower with both functional pistils and stamens
Sexual system	
Monomorphic	One kind of plant (floral morph) in the population
Hermaphrodite	Most commonly applied to plants with bisexual flowers, but all monomorphic populations consist of hermaphrodite individuals
Monoecious	Pistillate and staminate flowers on same plant
Gynomonecious	Both bisexual and pistillate flowers on same plant
Andromonoecious	Both bisexual and staminate flowers on same plant
Trimonecious	Bisexual, pistillate, and staminate flowers on same plant
Dimorphic	Two kinds of plants (floral morphs) in the population
Dioecious	One morph male (with staminate flowers only); the other female (with pistillate flowers only)
Gynodioecious	One morph female, the other hermaphrodite (with either bisexual flowers or both pistillate and staminate flowers)
Androdioecious	One morph male, the other hermaphrodite (as above)
Trimorphic	Three floral morphs in the population
Trioecious	Males, females, and hermaphrodites

Modified from Dellaporta and Calderon-Urrea 1993.

**Table 2:** List of dioecious species of angiosperms studied for dimorphic herbivore damage, and information on assessment of reproductive allocation, growth rate and resistance.

Species	Sex with greatest				Reference	Review
	Damage	Reproductive allocation	Growth rate	Resistance
Alismatales						
Araceae						
*Arisaema triphyllum *	M	nm	nm	nd (N, C:N, leaf total phenolics)	[24]	3
		F	F (total dry mass)		[25]	

Arecales						
Arecaceae						
*Chamaedorea alternans* (= *C. tepejilote*)	nd	nm	nm	nm	[26]	1
		F			[27]	
			M (leaf production)		[28]	

Asterales						
Asteraceae						
*Baccharis concinna *	nd	nm	nm	nm	[29]	
		nm	nd (leaf production) M (shoot length)	nm	[30]	3
*B. dracunculifolia *	nd	nm	nm		[31]	3
			nd (shoot length)		[32, 33]	
*B. halimifolia *	M, F, nd, depends on herbivore	nd (flowers/shoot)	M	F (resin)	[34]	1

Brassiclaes						
Capparaceae						
*Forchhammeria pallida *	nd	nm	nm	nm	[35]	3
Caryophyllaceae						
*Silene dioica *	M	M (during flowering)	F	nm	[36]	1
	F	nm	nd (length of infected shoots)	nm	[37]	3
Chenopodiaceae						
*Atriplex canescens *	F	nm	nm	nm	[38]	3
	M	nm	nm; nd (height, width, fresh weight in spring), F (FW in winter)	nm	[39]	4
		(F)			[40]; the species includes hermaphrodites	
*A. vesicaria *	F	nm	nm	nm	[41, 42]	1
Nyctaginaceae						
*Neea psychotrioides *	M	M	nd (stem production)	nm	[43]	3
Polygonaceae						
*Rumex acetosa *	M	?	?	?	T. Elmqvist unpublished data	1
*R. acetosella *	M, F, nd	nd	nm	nm	[44]	1
			F (ramet production)		[45]	

Fagales						
Myricaceae						
*Myrica gale *	M, nd	nm	nm	nm	L. Ericson unpublished data	1
	nd	nm	nm	F (1-digestibility), nd (phenolics, p-glycosides, tannins)	[46]	

Laurales						
Lauraceae						
*Lindera benzoin *	nd	nm	nd, M, depending on year	nm	[47]	1
		M (flowers/shoot), F (N and biomass)	M (plant volume)	F (phenolics on leaves, but nd on stems)	[48]	

Malpighiales						
Salicaceae						
* Populus tremula *	M	nm	nm	M (phenolics), nd (p-glycosides, tannins, digestibility)	[46]	1
* Salix caprea *	M,	nm	nm	F (1-digestibility), nd (phenolics, p-glycosides, tannins)	[46]	1
	nd		nd		[49]	
	nd	nm	nm	nm	[50]	4
* S. cinerea *	M, nd, varies by year	nm	nm	nm	[51]	1
			nd		[52]	
				nd (phenolic glycosides)	[53]	
* S. eleagnos *	M	nm	nm	nm	[50]	3
* S. fragilis *	nd	nm	nm	nm	[50]	4
* S. lanata *	nd	nm	nm	nm	[54]	3
* S. lasiolepis *	M (4 of 5 spp. of sawflies)		M (shoot length)	F (phenols, marginally significant)	[55]	1
	nd (miners, gallers)	nm	nm	nm	[56]	1
* S. myrsinifolia-phylicifolia *	M (at high plant density)	nm	nm	nm	[36]	1
	M	nm	nd (new shoots)	nm	[57]	1
	M (in high productivity habitat; decreases at higher herbivore pressure)	nm	nd (biomass)	nm	[49]	1
* S. pentandra *	M	nm	nm	F (phenolics) M (1-digestibility)	[46]	1
* S. purpurea *	nd	nm	nm	nm	[50]	4
* S. sericea *	M marginal	nm	nm	nm	[58]	3
			nd	nd	[53]	
* S. viminalis *	nd	nm	nd (regrowth after pruning)	nm	[59]	3
	nd	nm	nm	nm	[50]	4
* S. *x* rubens *	nd	nm	nm	nm	[50]	4

Pandanales						
Pandanaceae						
* Freycinetia arborea *	M	nm	nm	nm	[60]	2
* F. reineckei *	M	nm	nm	nm	[60]	2, 3

Rosales						
Eleagnaceae						
*Hippophae rhamnoides *	M ?	?	?	?	L. Ericson unpublished data	1
			M		[61]	
Rhamnaceae						
*Rhamnus alpinus *	nd			M (anthraquinones)	[62]	3
		F	nd if age < 10 y M if age > 10 y		[63]	
Rosaceae						
*Rubus chamaemorus *	M, nd	nm	nm	nd	[64]	1
		F but varies with fruit set			[65]	
Urticaceae						
*Urtica dioica *	M ?	?	?	?	T. Elmqvist unpublished data	1

Sapindales						
Sapindaceae						
*Acer negundo *	M	nm	M (growth rings)	nd (astringency, total phenols, nitrogen, toughness), F (index of defence)	[66]	1
			variable: F near streams; M away from streams		[67]	
			nd		[68]	
			F		[69]	
*Pistacia lentiscus *	nd	nm	nm	nd (N)	[70]	1
Simaroubaceae						
*Simarouba glauca *	M	nm	nm	two flavonoid compounds on female flowers not present in male flowers	[71]	3

F: female, M: male, nd: no statistically significant intersexual differences, nm: not measured, CT: condensed tannins, TNC: total non-structural carbohydrates, N: nitrogen content (herbivores usually attracted to greater concentrations).

1: Ågren et al. 1999 [72], Table 2.

2: Ågren et al. 1999 [72], Table 3.

3: Cornelissen and Stiling 2005 [73].

4: Not mentioned in any of 1–3 above.

**Table 3:** Studies of defence on dioecious species published after 2004, or published earlier but not mentioned in Ågren et al or Cornelissen and Stiling's reviews.

Species	Sex with greatest				Herbivores	Reference
	Damage	Reproductive allocation	Growth rate	Resistance
Arecales						
Arecaceae						
*Chamaedorea alternans (= C. tepejilote) *	M	F	F	F	Chrysomelid beetles	[74]
*C. pinnatifrons *	M	F	M	F	Chrysomelid beetles	[74]
*C. ernesti-augusti *	M	F	M	F	Chrysomelid beetles	[74]

Aquifoliales						
Anacardiaceae						
*Ilex glabra *	nd; marginally F after flowering	nd	nd	nm	lepidopteran larvae and leaf spot (fungal pathogens)	[75]

Sapindales						
Anacardiaceae						
*Sclerocarya birrea *	F	nd (wood/reproductive shoot)	nm	nd (wood density, branch breakability)	Elephants	[76]
*Spondias purpurea *	F	nm	nm	M (N, TNC)	Cerambycid beetle	[77]

Malpighiales						
Salicales						
*Salix discolor *	nd	nm	nm	M (mortality of herbivore)	Leaf galler	[78]
*S. polaris *	nm	F	nd	nd (phenolics, CT)	Reindeer	[79]
*S. arctica *	nd	nm	nd	nm	Muskox	[80]
*S. planifolia *	nd	F	nm	nm	Insects	[81]

Laurales						
Lauraceae						
*Lindera obtusiloba *	nd	nm	nm	nm	Unspecified	[82]
*L. praecox *	nd	nm	nm	nm	Unspecified	[82]
*L. umbellata *	nd	nm	nm	nm	Unspecified	[82]
*L. erythrocarpa *	nd	nm	nm	nm	Unspecified	[82]

Unplaced (Euasterids I)						
Hydrophyllaceae						
*Nemophila menziesii *	nd	nm	nm	nm	Larvae of lepidoptera (2 spp.) and coleoptera (1 sp.)	[83]

F: female, M: male, nd: no statistically significant intersexual differences, nm: not measured, CT: condensed tannins, TNC: total non-structural carbohydrates, N: nitrogen content (herbivores usually attracted to greater concentrations).

**Table 4:** Total number of species, number of dioecious species, proportion of dioecious species, and estimated 2% of dioecious species in the top 30 most species-rich families with a proportion of dioecious species greater than 0.5 (from unpublished data from S. Renner, University of Munich).

Family	Total species	Dioecious species	Proportion of dioecious species	2% of dioecious species
Arecaceae	815	778	0.955	16
Pandanaceae	777	777	1.000	16
Lauraceae	1123	776	0.691	16
Menispermaceae	577	577	1.000	12
Ebenaceae	487	487	1.000	10
Anacardiaceae	594	439	0.739	9
Salicaceae	436	435	0.998	9
Myristicaceae	367	365	0.995	7
Clusiaceae	590	365	0.619	7
Restionaceae	387	364	0.941	7
Aquifoliaceae	400	300	0.750	6
Smilacaceae	215	205	0.953	4
Cucurbitaceae	390	197	0.505	4
Flacourtiaceae	209	192	0.919	4
Burseraceae	234	175	0.748	4
Cecropiaceae	184	174	0.946	3
Thymelaeaceae	236	119	0.504	2
Vitaceae	155	118	0.761	2
Loranthaceae	147	114	0.776	2
Meliaceae	181	105	0.580	2
Theaceae	155	94	0.606	2
Proteaceae	84	84	1.000	2
Hydrocharitaceae	123	75	0.610	2
Monimiaceae	108	74	0.685	1
Rhamnaceae	140	71	0.507	1
Nepenthaceae	70	70	1.000	1
Siparunaceae	93	68	0.731	1
Myricaceae	52	51	0.981	1
Chloranthaceae	57	51	0.895	1
Casuarinaceae	96	51	0.531	1

Total	9482	7751		155
